# Management of Myomectomy Scar Pregnancy: A Scoping Review

**DOI:** 10.3390/medicina61050817

**Published:** 2025-04-29

**Authors:** Felice Sorrentino, Lorenzo Vasciaveo, Francesca Greco, Elisa Giansiracusa, Francesco D’Antonio, Alessandro Lucidi, Andrea Etrusco, Antonio Simone Laganà, Guglielmo Stabile, Luigi Nappi

**Affiliations:** 1 Department of Medical and Surgical Sciences, Institute of Obstetrics and Gynecology, University of Foggia, 71121 Foggia, Italy; l.vasciaveo@gmail.com (L.V.); grecofrancesca1989@gmail.com (F.G.); elisag98@live.it (E.G.); guglielmost@gmail.com (G.S.); luigi.nappi@unifg.it (L.N.); 2 Centre for Fetal Care and High-Risk Pregnancy, Department of Obstetrics and Gynaecology, University of Chieti, 66100 Chieti, Italy; dantoniofra@gmail.com (F.D.); lucidi.alex@gmail.com (A.L.); 3 Unit of Obstetrics and Gynecology, “Paolo Giaccone” Hospital, Department of Health Promotion, Mother and Child Care, Internal Medicine and Medical Specialties (PROMISE), University of Palermo, 90127 Palermo, Italy; etruscoandrea@gmail.com (A.E.); antoniosimone.lagana@unipa.it (A.S.L.)

**Keywords:** myomectomy, scar pregnancy, leiomyoma, PAS disorders

## Abstract

*Background*: Ectopic pregnancy (EP) is defined as the implantation of an embryo outside the uterine cavity, which can lead to high morbidity and mortality if not diagnosed and treated promptly. A rare form of EP is myomectomy scar pregnancy (MSP), where the embryo implants in a scar from a prior myomectomy. Due to its rarity, MSP presents unique diagnostic and therapeutic challenges. This scoping review aims to map the existing literature on MSP to better understand the diagnostic strategies, management options, and clinical outcomes associated with this condition, and to identify gaps in current research. *Methods*: We conducted a scoping review by searching databases such as PubMed, Scopus, Web of Science, and MEDLINE for studies published between 2003 and 2023. Keywords used in combination included “myomectomy scar pregnancy”, “scar pregnancy”, “leiomyoma”, “uterine myomectomy”, “PAS disorders”, “placenta previa”, and “placenta accreta”. Studies were screened for relevance and eligibility by two independent reviewers. Data were extracted from case reports, retrospective studies, and reviews discussing MSP. *Results*: From an initial set of 111 studies, 28 papers met the inclusion criteria, comprising 4 retrospective studies and 24 case reports. A total of 44 cases of MSP were analyzed. The majority of diagnoses were made through ultrasound, with magnetic resonance imaging (MRI) used in more complex cases. Surgical interventions, primarily cesarean sections and myometrial repairs, were the most common treatments, while medical therapy with methotrexate was less frequently applied. *Conclusions*: This scoping review highlights the challenges of diagnosing and managing MSP due to its rarity. Although surgical management remains the primary approach, there is a lack of consensus on the optimal treatment for different clinical scenarios. Further research is needed to establish standardized diagnostic and therapeutic protocols for MSP and to evaluate the long-term outcomes of affected patients.

## 1. Background

Leiomyomas, also known as uterine fibroids or myomas, are benign tumors of the smooth muscle of the uterus that affect women during their reproductive years [1]. They are extremely heterogeneous in terms of pathophysiology, size, location, and clinical symptoms [2,3]. Uterine fibroids, especially those of submucosal and intramural origin, can also be associated with reproductive problems such as infertility, recurrent miscarriages, and unfavorable obstetric outcomes [4,5,6]. Treatment options for fibroids include surgery, a pharmacological approach, and interventional radiology. In the presence of fibroids and infertility, surgical treatment is preferable to drug therapy [7]. Minimally invasive surgery, in particular, offers significant advantages and is currently the preferred surgical option [8]. For subserous–intramural myomas, laparoscopic and robotic removal offer the best surgical solution [9]. Hysteroscopic removal is recognized as the most effective gold standard for the management of FIGO classes 1 and 2 submucosal fibroids [10].

A myomectomy scar pregnancy, a type of ectopic scar pregnancy, is a rare complication in women who undergo surgical treatment.

“Ectopic scar pregnancy” is defined as a rare form of abnormal implantation of an embryo in the fibrous tissue of the myometrium caused by a scar, resulting from a previous uterine surgical procedure, such as cesarean section, dilation and curettage, abnormal placentation, myomectomy, metroplasty, and manual placental delivery [11]. Two different types of scar ectopia have been identified: Type I is caused by embryo implantation into a previous scar with spread into the cervical space (if from a previous cesarean section) or into the uterine cavity; Type II is caused by deep embryo implantation into the scar with infiltrative growth into the uterine myometrium and serosal surface of the uterus, which can lead to uterine rupture and life-threatening massive hemorrhage in the first trimester of pregnancy [12]. Pregnant women with previous uterine scarring have an increased risk of morbidity. Myomectomy scar pregnancy, as seen in other scar pregnancies, usually shows no specific symptoms, so its diagnosis can be easily overlooked, leading to a high risk of possible life-threatening hemorrhage during pregnancy as well as uterine rupture, disseminated intravascular coagulation, and even death.

Myomectomy scar pregnancy is an extremely rare pathological condition, even though in recent years a trend of pregnancies in older maternal age has been detected, determining a continuously increasing incidence of this condition due to the increasing number of pregnancies following the widespread use of laparotomic (LPT) and laparoscopic (LPS) myomectomy [13].

The aim of this review is to research and investigate the clinical and therapeutic management of myomectomy scar pregnancy in current scientific literature.

## 2. Materials and Methods

### 2.1. Research Strategy

This scoping review, which explores pregnancies implanted in a scar from a previous myomectomy, was carried out by searching the following databases: PubMed, Scopus, Web of Science, and MEDLINE. We used the following keywords alone and in combination: “myomectomy scar pregnancy”, “scar pregnancy”, “leiomyoma”, “uterine myomectomy”, “PAS disorders”, “placenta previa”, and “placenta accreta”. We included studies published in English from 2003 to 2023 to provide a broad overview of the available literature. The review aimed to map current knowledge and identify gaps for future research. Studies were screened for relevance based on the title and abstract by two independent reviewers. Articles were then assessed for eligibility through full-text review. Any discrepancies in data interpretation or classification between reviewers were resolved through discussion and consensus. In cases of persistent disagreement, a third senior reviewer was consulted to reach a final decision. This approach ensured consistency and accuracy in data extraction and analysis.

### 2.2. Inclusion Criteria

- Studies reporting on myomectomy scar pregnancies (MSP);
- Case reports, retrospective studies, and reviews discussing the diagnosis, management, and outcomes of MSP;
- Articles published in English within the defined period.

### 2.3. Exclusion Criteria

- Studies not specifically addressing MSP;
- Articles not in English or lacking available translations;
- Papers with incomplete or low-quality data.

Due to the variability in the data, a formal statistical meta-analysis was not feasible. Instead, a narrative synthesis was conducted. Data were grouped and analyzed descriptively based on key clinical variables, including maternal age, type of myomectomy, pregnancy type (spontaneous or ART), gestational age at diagnosis, diagnostic modality, type of intervention, and clinical outcomes. Themes and trends were identified across the included case reports and retrospective studies to provide an overview of current management practices and highlight recurring complications. Findings were summarized in descriptive tables to highlight similarities and differences in management and clinical outcomes.

## 3. Results and Discussion

**Figure 1:**
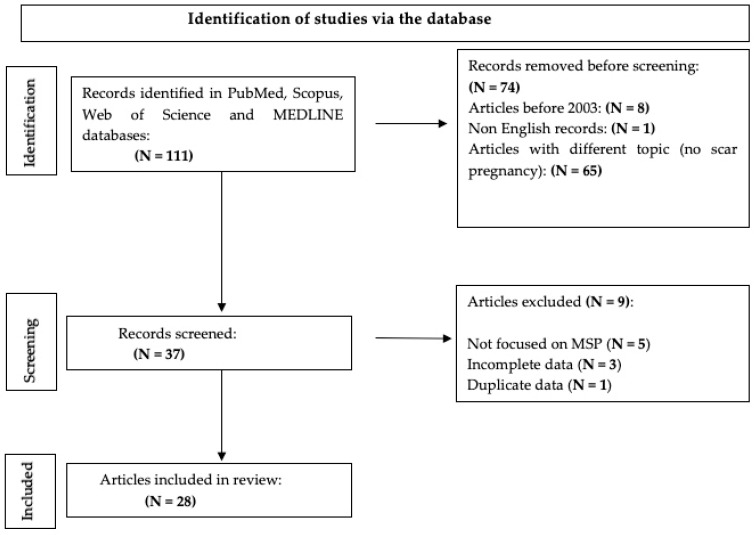
PRISMA flow diagram.

In total, 111 studies were found, but only 28 papers were analyzed, in particular 4 retrospective studies and 24 case reports. Specifically, eight papers were excluded because they were published before 2003. One paper was not included because it was written in Japanese. In total, 65 studies were not included because they did not deal with scar pregnancies. Finally, nine papers were not included because, although they dealt with scar pregnancies, they did not occur after myomectomy as a previous operation [Figure 1].

Data regarding maternal age, type of myomectomy surgery, whether the patients had a spontaneous or Assisted Reproductive Technology (ART) pregnancy, the exact week of gestation when the diagnosis of scar pregnancy was made, the proposed therapy for the pregnant women, maternal outcome, and possible complications were fully analyzed. Of the 28 papers included in the review, 4 were retrospective studies, while 24 were case reports, allowing 44 cases of myomectomy-related scar pregnancy to be analyzed, as shown in Table 1.

The average age of the pregnant women was 36 years (age range: 19–48 years). More than one-third of the patients had a history of primitive infertility for which they required ART after myomectomy (14 out of 37 cases). The diagnosis was mainly made by ultrasound, a method that is widely used due to its low cost, repeatability, and reproducibility. For better case management, magnetic resonance imaging was used as the imaging technique of choice to more accurately assess the location of the pregnancy and the anatomical connections to other nearby organs. In more than half of the cases, the uterine fibroids were removed by hysteroscopy (19 out of 36 cases) (Table 2).

Among the 44 cases of scar pregnancy, 13 of them had PAS disorder, and 3 cases showed a severe condition (placenta percreta).

**Figure 2:**
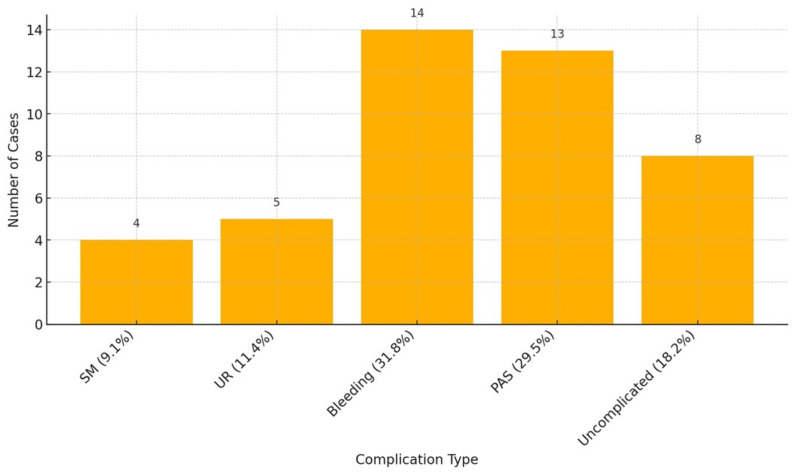
Complications reported in the included studies. (SM: Spontaneous Miscarriage; UR: Uterine rupture; BP: bleeding during pregnancy; PAS: placenta accreta spectrum; UP: uncomplicated pregnancy). NB “Uncomplicated pregnancy” refers to cases in which no maternal or fetal complications were explicitly reported in the original publication. Subclinical or minor complications may not have been assessed or documented.

In this review, we included 28 studies from 2006 to 2022, describing all reported complications (Figure 2).

The complications considered were spontaneous miscarriage (SM), uterine rupture (categorized as before or after 24 weeks of gestation), bleeding during pregnancy, placenta accreta spectrum, severe placenta accreta (placenta percreta), and uncomplicated pregnancy. Uncomplicated pregnancy refers to cases where no complications were explicitly reported in the original study. Subclinical findings may not have been assessed or mentioned). In the first three columns are shown the general characteristics of each study: the first author, the study’s year of publication, and the number of cases. Key study characteristics are organized into columns, and the number of cases included in each study is reported accordingly. Above all, 4 cases of SM are reported (9.1%), 5 cases of uterine rupture (11.4%), 14 cases of bleeding during pregnancy (31.8%), 13 cases of PAS disorders (29.5%), and 8 cases of uncomplicated pregnancy (18.2%). In most of the uneventful cases, MSP was diagnosed during pregnancy through ultrasound or MRI. However, in a few instances, the diagnosis was made retrospectively during cesarean section or histopathological examination at delivery, when abnormal placental attachment to a previous myomectomy site was observed.

The uterine rupture was classified into before and after 24 weeks of gestation, showing 2 cases of uterine rupture before 24 weeks (40%) and 3 cases after 24 weeks (60%). The biggest study by Kasuga et Al, published in 2020, collected 14 cases, and 50% were uncomplicated pregnancies. In this study, the complication in 28.6% is the presence of bleeding during pregnancy, and only one case was reported as a severe case of placenta accreta disorder (Placenta Percreta).

In summary, the following has been demonstrated: several studies reported no cases of uterine rupture; some cases reported bleeding during pregnancy; placenta accreta spectrum was noted in multiple studies, with some cases classified as severe placenta percreta; a few cases involved spontaneous miscarriage; some studies reported completely uncomplicated pregnancies.

Some patients (5 out of 44 cases) were found to have a uterus rupture, identified by using ultrasound or by direct evaluation through laparotomy or laparoscopy. The treatment of choice for scar pregnancies is surgical technique; only rarely is drug therapy or a “wait and see” approach considered a valid treatment option. The correction of myometrial scars and the removal of ectopic pregnancies is mainly performed by cesarean section or laparotomy. Unfortunately, there were a considerable number of cases in which it was necessary to carry out a hysterectomy (10 out of 44 cases). Only a few cases of scar pregnancy were treated with drug therapy (methotrexate), and often they even require supportive surgical therapy (3 out of 44 cases) (Table 3).

Of the 44 cases examined, 4 patients also have a history of previous cesarean section (9%). The location and size of myomas cannot be statistically evaluated, as many authors have not reported these characteristics in their case reports. The location and size of myomas are crucial factors for understanding the risk and management of myomectomy scar pregnancies (MSP). However, the data available in this review reveal a significant lack of standardized reporting in many case reports. Among the 44 cases analyzed, only a portion of the authors provided detailed information on the localization and size of myomas. Specifically, submucosal myomas were the most frequently involved (21/44, 47% of cases), followed by intramural (8/44, 18%) and cervical myomas (2/44, 4.5%). The mean size of myomas undergoing myomectomy was estimated at 6 cm, with a median of 6.6 cm (Table 4).

This variability in data reporting limits the ability to perform a more detailed analysis of the correlation between myoma characteristics and the incidence or risk of MSP. Future studies should prioritize the systematic collection of this information, using consistent criteria to define localization (e.g., FIGO classification) and to measure size (maximum diameter). Standardized reporting would facilitate the identification of risk patterns and support the development of more effective clinical protocols for managing these patients.

## 4. Discussion

The findings of this scoping review directly reflect the study’s aim to map the current literature on MSP, clarify clinical characteristics, and identify management trends and complications. In doing so, our analysis highlights key challenges such as a lack of standardized treatment approaches and significant variability in case documentation, which align with the gaps identified in the introduction. Published data show a higher number of pregnancies in surgically manipulated uteri, where complications (placenta previa, spontaneous uterine rupture, uterine dehiscence with or without placental invasion, uterine hemorrhage, and spontaneous miscarriage) are common [42]. The surgeon’s skills and experience are essential to ensure good repair of the myometrium after myoma removal, leaving a strong and permanent scar. Connective tissue cannot fully replace myometrial function due to structural differences, limiting its ability to actively participate in pregnancy-related changes. Therefore, it is imperative to perform a careful closure of the uterine scar to enable a safe pregnancy [43]. Pregnancies after myomectomy require careful clinical monitoring, especially because several studies have shown that myomectomy scars (0.6%) have a higher risk of rupture during pregnancy than cesarean section scars (0.3%) and septoplasty scars (0.02%) [44,45].

Although there are numerous studies in the literature on pregnancy complications in pregnant women after a previous myomectomy [46,47], there are very few papers dealing with rare complications such as MSP, which is related to the implantation of the placenta at the site of the previous myomectomy. Although we consider it a quite rare event, this type of placenta that pathologically attaches in the uterine cavity belongs to the well-known group of placenta accreta spectrum (PAS) [48]. Placental disorders are often associated with uterine wall surgery, but the aetiopathogenesis of this relationship is not yet fully understood. A disruption of the endometrial-myometrial interface caused by uterine surgery is probably the cause of the absent or incomplete formation of Nitabuch’s fibrinoid layer. This layer is responsible for normal placentation, and its absence leads to abnormal placentation. In placenta accreta, massive hemorrhage at birth is the main clinical problem. Prenatal detection of PAS disorders is crucial, as timely referral of suspected cases to specialized referral centers with multidisciplinary teams ready to treat these complex cases on an ongoing basis is key to reducing perinatal and maternal morbidity and mortality [49].

A retrospective Latin American study reported that 40% of the 52 PAS-related maternal deaths were not diagnosed prenatally, and almost 46% of them were not investigated immediately before delivery in a PAS referral hospital. According to the authors, all deaths were potentially preventable by interventions of low to moderate complexity in 77% of cases [50]. The clinical practice and management of scar pregnancies are based on the patient’s clinical condition, gestational age, the number of previous pregnancies, the use of ARTs, the experience of the medical center, and the patient’s desire for fertility—crucial factors that must be taken into account in order to achieve the best outcome. Early diagnosis of these disorders is important for doctors and patients when making decisions. High-risk patients with clinical and radiological findings suggestive of placenta accreta should undergo accurate prenatal monitoring, as they have a higher likelihood of prolonged hospitalization, a higher risk of preterm delivery, and an increased risk of hysterectomy by cesarean section. Treatment options include surgical resection, hysteroscopic excision, hysteroscopic laser surgery, local injection of potassium chloride, and methotrexate [23,51,52,53]. The main advantage of surgery is its definitive approach, whereas drug therapy can lead to slow and/or incomplete resolution, resulting in uncertainty and an increased risk of uterine rupture. However, in some specific cases, medical treatment of scar pregnancies following hysteroscopy has been successful, with women retaining fertility [14,15,54,55,56]. Due to the high risk of uterine rupture, waiting is not considered a valid alternative. Most of the treatments described in the included studies were reactive, and implemented in response to complications such as uterine rupture, hemorrhage, or suspected PAS disorders. This reactive approach reflects both the rarity and the diagnostic challenges of MSP, which often prevent early recognition and structured planning. The predominance of emergency procedures, including hysterectomy in severe cases, highlights the urgent need for developing standardized protocols that support earlier detection and individualized, preemptive management strategies to reduce maternal morbidity and preserve fertility when possible.

## 5. Conclusions

This scoping review has mapped the existing literature on myomectomy scar pregnancies (MSP), highlighting the challenges in diagnosing and managing this rare condition. Surgical management remains the primary therapeutic approach; however, there is currently no consensus on the optimal strategy, particularly given the variability of clinical presentations. The review also identified significant gaps in the literature, especially regarding long-term outcomes and the effectiveness of non-surgical approaches. Clinically, early and accurate diagnosis—preferably in the first trimester—combined with individualized treatment planning is crucial to reduce the risk of emergency procedures such as uterine rupture or hysterectomy. Increased awareness among obstetricians and the routine consideration of MSP in patients with a surgical uterine history are essential steps to improve outcomes. Future research should focus on the following:

- establishing standardized diagnostic criteria and reporting formats, including FIGO classification for myoma location and size;
- developing evidence-based treatment algorithms that differentiate between stable and emergency cases;
- creating multicenter registries to gather prospective data;
- evaluating the long-term reproductive outcomes of different treatment modalities, including conservative and fertility-sparing approaches.

Progress in these areas will strongly depend on collaborative efforts and structured data collection, which are essential to improving the understanding and management of MSP and optimizing patient-centered care.

## 6. Limits of the Paper

This review has several limitations that should be acknowledged. Firstly, some of the selected studies presented missing or incomplete data, particularly regarding patients’ age, diagnostic methods, and surgical details. In particular, important clinical variables such as myoma size and anatomical location were frequently omitted or inconsistently reported, which limited the depth of analysis and reduced our ability to identify correlations with clinical outcomes. This inconsistency may introduce bias and reduce the overall comparability of the included cases. Secondly, the review includes only articles published in English, which may have led to the exclusion of potentially relevant studies written in other languages, introducing a possible language bias. Moreover, due to the rarity of myomectomy scar pregnancy (MSP) and the heterogeneity of the available data—which mostly derive from isolated case reports and small retrospective series—it was not possible to perform a formal quantitative synthesis or meta-analysis. This restricts the ability to draw statistically supported conclusions and limits the generalizability of our findings. Future research should aim to standardize reporting criteria and develop multicenter registries to enable more robust analyses.

## Figures and Tables

**Table 1:** Data from included studies.

Author	Year	Country	Study Design	Period Considered	GA at Diagnosis	Intervention	Cases (n)	Preview Myomectomy Surgical Procedures	Age	Diagnostic Exam	Spontaneous Pregnancy or ART
Ballas [14]	2012	USA	retrospective	2004–2011	35 w; 40 w	CS + hysterectomy	2	A: HSC; B: HSC	na	na	na
Kasuga [15]	2020	Japan	retrospective	2012–2019	20	observation	14	14 HSC	36 (28–41)	14 TA US	3 ART 11 spontaneous
Kocher [16]	2017	Carolina	case report	2017	20 w	CS + hysterectomy	1	na	37	US + MRI	ART
Matheisen [17]	2015	USA	case report	2013	26 w	CS + hysterectomy	1	HSC	34	MRI	spontaneous
Lo [18]	2015	Hong Kong	case report	2015	20 w	CS	1	LPS	41	US	spontaneous
Saleh [19]	2022	Germany	case report	2022	27 w + 4	CS + hysterectomy + bowel resection	1	na	47	MRI	na
Tanaka [20]	2016	Japan	case report	2016	39 w	hysterectomy	1	HSC	48	US	ART
Takeda [21]	2022	Japan	case report	2021	18 w, 14 w	CS + hysterectomy	2	2 LPS	37; 38	2 MRI	ART
Zhu [22]	2020	China	case report	2019	6 w	LPT surgical removal + metotrexate	1	LPS	26	MRI	spontaneous
Park [23]	2006	Korea	case report	2006	7 w	LPS surgical removal	1	LPT	35	US	ART
Wong [24]	2010	Australia	case report	2003	na	metotrexate	1	na	33	US	spontaneous
Ishiguro [25]	2018	Japan	case report	2018	8 w	LPS surgical removal	1	LPS	41	US	ART
Paul [26]	2018	India	case report	2018	9 w	LPS surgical removal	1	LPS	31	US	spontaneous
Bannon [27]	2013	USA	case report	2013	10 w	LPS surgical removal + metotrexate	1	LPT	27	US + TC	spontaneous
Vagg [28]	2018	Australia	case report	2018	12 w	hysterectomy	1	LPT	34	US + MRI	spontaneous
Liu [29]	2019	China	retrospective	2018	na	HSC + LPS	1	na	34	CEUS	na
Li [30]	2018	Singapore	case report	2018	9 w	LPS surgical removal	1	LPS	40	US	na
Dutta [31]	2020	India	retrospective	2012–2019	na	LPT surgical removal	1	na	19	na	spontaneous
Al-Serehi [32]	2008	Canada	case report	2008	34 w	CS + hysterectomy	1	na	48	na	ART
Matsunaga [33]	2015	Japan	case report	2015	33 w; 37 wks	CS + myometral repair	2	2 LPS	39; 37	2 US	spontaneous
Fukutani [34]	2017	Japan	case report	2017	33 w	CS + myometral repair	1	LPS	35	MRI	ART
Kuwata [35]	2011	Japan	case report	2011	34 w	CS + myometral repair	1	LPT	38	TC	spontaneous
Bejarano [36]	2021	USA	case report	2019	37 w	CS + myometral repair + uterine artery embolization + HSC + D&C	1	LPS	41	TV US	ART
Agarwal [37]	2017	India	case report	2017	9	D&C + LPS	1	LPS	28	TV US + MRI	ART
Bouzari [38]	2010	Iran	case report	2010	26	LPT + myometral repair	1	na	28	US	spontaneous
Zhang H [39]	2021	China	case report	2021	4 wks	LPT surgical removal	1	HSC	38	US + MRI	ART
Kandaswami [40]	2022	India	case report	2015	4 wks	metotrexate + LPS	1	LPS	45	US	ART
Hudecova [41]	2018	Czech Republic	Case report	2017	12 wks	LPT	1	LPS	33	US + MRI	spontaneous

US: ultrasound; MRI: magnetic resonance imaging; TV US: transvaginal ultrasound; ART: Assisted Reproductive Technology; CS: cesarean section; HSC: hysteroscopy; LPS: laparoscopy; LPT: laparotomy; na: not available as not reported in the source article.

**Table 2:** Surgical and diagnostic characteristics of 44 myomectomy scar pregnancies.

	Total Cases	Cases (%)
Study design	44	
- Retrospective;		18 (40.9%)
- Case report.		26 (59.1%)
Age (mean ± SD)	36 ± 6.7 (range 19–48 years)	
Diagnostic exam	40	
- US;		27 (67.5%)
- MRI;		11 (27.5%)
- CT.		2 (5%)
Pregnancy	39	
- Spontaneous;		24 (61.5%)
- ART.		15 (38.5%)
Myomectomy surgical procedures	37	
- Hysteroscopy;		19 (51.3%)
- Laparotomy;		4 (10.8%)
- Laparoscopy.		14 (37.9%)

US: ultrasound; MRI: magnetic resonance imaging; CT: Computed Tomography; ART: Assisted Reproductive Technology.

**Table 3:** Intervention types in myomectomy scar pregnancy (MSP).

Intervention Type	Number of Cases (n)	Percentage (%)
Cesarean section + uterine repair	10	22.7
Vaginal delivery	9	20.5
Hysterectomy	10	22.7
Laparotomy + uterine repair	4	9.1
Laparoscopy + uterine repair	4	9.1
Methotrexate (medical treatment only)	4	9.1
Methotrexate + surgical approach	3	6.8
Combined therapies	3	6.8

Methotrexate has often been used in a single injection in local or systemic administration, with MSP situated in the uterine posterior wall.

**Table 4:** Characteristics of myomas.

	Cases	%
Previous Cesarean section	4	9%
Localization of myoma	21 Submucosal	47%
	8 Intramural	18%
	2 cervical	4.5%
Myomas size	Mean 6 cm	Median 6.6 cm

## Data Availability

All data are presented in the present manuscript
